# Interfacing broadband photonic qubits to on-chip cavity-protected rare-earth ensembles

**DOI:** 10.1038/ncomms14107

**Published:** 2017-01-16

**Authors:** Tian Zhong, Jonathan M. Kindem, Jake Rochman, Andrei Faraon

**Affiliations:** 1 T. J. Watson Laboratory of Applied Physics, California Institute of Technology, 1200 E California Boulevard, Pasadena, California 91125, USA

## Abstract

Ensembles of solid-state optical emitters enable broadband quantum storage and transduction of photonic qubits, with applications in high-rate quantum networks for secure communications and interconnecting future quantum computers. To transfer quantum states using ensembles, rephasing techniques are used to mitigate fast decoherence resulting from inhomogeneous broadening, but these techniques generally limit the bandwidth, efficiency and active times of the quantum interface. Here, we use a dense ensemble of neodymium rare-earth ions strongly coupled to a nanophotonic resonator to demonstrate a significant cavity protection effect at the single-photon level—a technique to suppress ensemble decoherence due to inhomogeneous broadening. The protected Rabi oscillations between the cavity field and the atomic super-radiant state enable ultra-fast transfer of photonic frequency qubits to the ions (∼50 GHz bandwidth) followed by retrieval with 98.7% fidelity. With the prospect of coupling to other long-lived rare-earth spin states, this technique opens the possibilities for broadband, always-ready quantum memories and fast optical-to-microwave transducers.

## Abstract (web summary)

Solid-state emitters enable broadband quantum information storage, but they are affected by decoherence resulting from inhomogeneous broadening. Here the authors suppress this effect via cavity protection at the single photon level in an ensemble of rare-earth ions coupled to a nanophotonic resonator.

Ensembles of rare-earth ions doped in crystals exhibit outstanding quantum coherence properties and large inhomogeneous linewidths^1^ that are suitable for quantum information transfer with broadband photons in high-speed optical quantum communication networks^2^,^3^,^4^. They are used in state-of-the-art optical quantum memories with potential for microwave storage^5^,^6^,^7^,^8^,^9^,^10^,^11^ and are promising candidates for optical-to-microwave quantum transduction^12^,^13^. One major challenge towards broadband quantum interfaces based on solid-state emitters is that information stored in the collective excitation of the ensemble quickly decoheres due to inhomogeneous broadening. To restore the optical coherence, protocols based on spectral hole burning techniques like atomic frequency comb (AFC)^7^,^8^ and controlled reversible inhomogeneous broadening^9^ have been perfected. Although effective, these protocols involve long (hundreds of milliseconds) and complex preparation procedures that generally limit the interface bandwidth. Recently, it was proposed^14^,^15^ that ensemble decoherence can be suppressed via strong coupling to a cavity. This phenomenon, called cavity protection, has been experimentally observed, though not in full effect, in the microwave domain with a nitrogen vacancy spin ensemble^16^. Prior experiments reported polariton linewidth narrowing in the quantum well structures^17^, but it is not yet conclusive that those effects were results of cavity protection.

Here, we demonstrate strong cavity protection against decoherence in the optical domain using a dense ensemble (a few millions) of neodymium (Nd) atoms coupled to a nanophotonic cavity. Exploiting the protected mapping of photonic qubits to atomic super-radiant excitations, we realize an efficient quantum light-matter interface with ∼50 GHz bandwidth that could find applications in future quantum networks.

## Results

### Conditions for cavity protection

**Figure 1:**
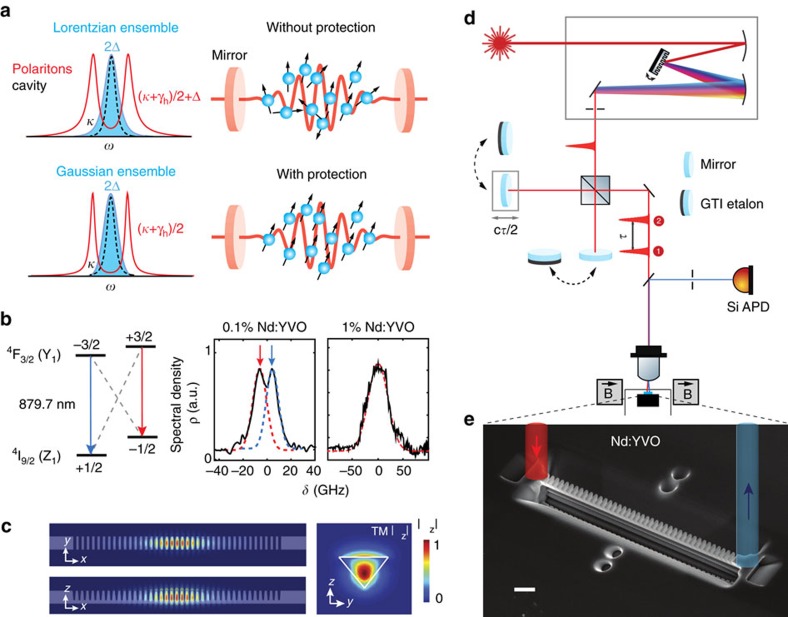
Schematics of the cavity protection effect. (**a**) Conceptual illustration of cavity protection for an ensemble coupled to a cavity mode. For a Lorentzian ensemble (upper), the polaritons are not protected and undergo dephasing (linewidth broadening) due to inhomogeneous broadening Δ. A Gaussian ensemble (lower) can be fully protected with the collective super-radiant excitation free of such dephasing, and the polariton linewidths do not depend on Δ. Arrows represent the phasor of each atomic dipole. (**b**) Energy levels and transitions (dotted lines are forbidden) for Nd (left). Measured absorption spectra for 0.1 and 1% Nd:YVO (right). Two Zeeman split sub-ensembles are resolved in the 0.1% sample. (**c**) Simulated TM resonance mode profiles of the triangular nanobeam resonator. (**d**) Experimental set-up. Two pico-second pulses were transmitted through the cavity and the output signal was integrated on a Si APD photon counter. (**e**) Scanning electron microscope image of the device and schematics of input and output optical coupling. Scale bar, 1 μm.

The dynamics of a coupled cavity-ensemble system are described by the Tavis–Cummings Hamiltonian^18^. The interaction term reads *H*_int_=*i*ℏΩ(*S*^−^*a*^†^−*S*^+^*a*) where *a*^†^ and *a* are creation and annihilation operators of the cavity mode, and the collective spin operators 
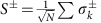
 act on *N* atoms each of frequency *ω*_*k*_. Ω denotes a collective coupling strength 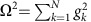, which scales up the single atom coupling *g*_*k*_ by 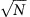. On resonance, the coupled system exhibits two bright polariton states with equal mix of atomic and photonic components detuned by ±Ω from the mean ensemble frequency. The polaritons decay via radiative emission and decohere by coupling to dark subradiant states that overlap spectrally with the ensemble^14^,^15^,^19^. The dark-state coupling critically depends on the energy separation between the polaritons and the subradiant states, and also on the specific profile of the inhomogeneous spectral distribution 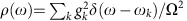 (refs 14, 15). In the limiting case of a Lorentzian distribution, considerable damping given by the width of the inhomogeneous broadening persists even with an infinite Ω. When the spectral distribution exhibits a faster-than-Lorentzian decay (for example, Gaussian), the damping of the coherent Rabi oscillation is diminished at increasing Ω—the system becomes ‘cavity protected' as conceptually illustrated in Fig. 1a. In this case, the atomic component of the polariton is purely the symmetric super-radiant state^20^.

### Observation of cavity protection in Nd ensembles

We probe the cavity protection regime in optical nanocavities based on our triangular beam design^21^,^22^ fabricated in Nd-doped yttrium vanadate (YVO) crystals (United crystals; Fig. 1e). The cavities have fundamental TM mode resonances with measured quality factor Q of 7,700 (*κ*∼2*π* × 44 GHz is the energy decay rate, that is, full-width at half-maximum (FWHM) is 44 GHz) and 17,000 (*κ*∼2*π* × 20 GHz, FWHM=20 GHz) in 0.1 and 1% Nd:YVO, respectively. A simulated mode volume *V*_mode_=1(*λ*/*n*)^3^=0.063 μm^3^ estimates *N*∼10^6^ (10^7^) ions in the 0.1% (1%) cavity. The resonance wavelengths are close to the ^4^*I*_9/2_(*Y*_1_)−^4^*F*_3/2_(*Z*_1_) transition of Nd^3+^ at 879.7 nm. The devices were cooled down to 3.6 K (Montana Instruments Cryostation) and a magnetic field of 500 mT was applied perpendicular to the YVO *c*-axis. In 0.1% Nd:YVO, the optical coherence time is *T*_2_=390 ns (measured via photon echoes), corresponding to a single emitter homogeneous linewidth *γ*_h_/2*π*=1/*πT*_2_=0.82 MHz. In 1% Nd:YVO, we measured an upper bound of *γ*_h_/2*π*≤40 MHz via transient hole burning. The B field caused a Zeeman splitting of the *Y*_1_, *Z*_1_ states into four levels (Fig.1b). For this field orientation, cross-transition probabilities are minimized^23^. Therefore the system can be viewed as two independent distributions of emitters separated by 17 GHz (shown as resolved absorption lines in 0.1% but not in 1% device) both coupled to the cavity with similar strengths. To capture the spectral shape of the distribution, a *q*-Gaussian function was used to fit each transition^16^, yielding a shape parameter *q*=1.01 (1 for Gaussian, 2 for Lorentzian) for the 0.1% ensemble. Each Zeeman branch has a FWHM of 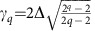
=2*π* × 8.3 GHz with Δ/2*π*=5.0 GHz (Δ/*π* represents the FWHM for a Lorentzian distribution). The total FWHM of the ensemble including both branches is 24 GHz. The 1% ensemble exhibits an asymmetric distribution with 76 GHz FWHM. However, it cannot be fitted well with any common functions because at this concentration the ions exhibit various interactions between themselves and with crystalline defects that lead to satellite lines^24^.

**Figure 2:**
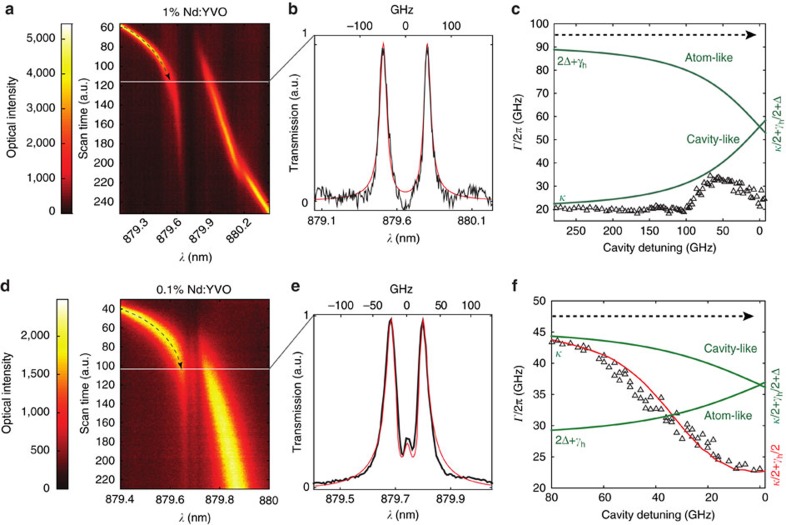
Cavity protection of Nd ensembles against decoherence. (**a**,**d**) Cavity transmission spectra while tuning its resonance across the inhomogeneous Nd transition. (**b**,**e**) On-resonance transmission spectra showing two bright polaritons. Red curves are the theoretical fit assuming Gaussian ensembles. (**c**,**f**) Experimental decay rates extracted from the left anti-crossing trajectory (dotted arrows) in **a** and **d**, respectively, as a function of detuning. In **c** (1% Nd:YVO), the polariton (21 GHz) is significantly narrower than the initial inhomogneous broadening (76 GHz), but it is not reaching the full protection limit likely due to an asymmetric, non-Gaussian ensemble shape. In **f** (0.1%), the polariton linewidth decreases rapidly towards resonance to the full protection limit (*κ*/2+*γ*_h_/2). Red curve plots the theoretial decay for a Gaussian distribution. Green curves show the theoretical decays assuming a Lorentzian ensemble of the same Δ.

To achieve strong coupling, the cavity resonance was tuned towards longer wavelengths by gas condensation^21^ while the transmission from a broadband superluminescent input was recorded in Fig. 2a,d using a spectrometer (Supplementary Movies 1 and 2). The on-resonance spectra (Fig. 2b,e) reveal two bright polariton peaks with a Rabi splitting of Ω_R_/2*π*=110 GHz and 48 GHz, for the 1 and 0.1% device, respectively. In Fig. 2e, a middle peak is present in between the polaritons because the cavity coupled simultaneously to two Zeeman branches with a resolved splitting (Supplementary Notes 1 and 2). The decay rates Γ(*δ*) were determined from the FWHM linewidth of the left polariton peak and are plotted against the cavity-ensemble detuning *δ*=*ω*_*c*_−*ω*_*a*_ in Fig. 2c,f as black triangles. The data corresponds to the left anti-crossing trajectory in Fig. 2a,d as the cavity shifted from shorter wavelengths towards the atomic resonance.

The phenomenon of cavity protection can be observed in both concentration samples, as the on-resonance Γ is considerably narrower than the Lorentzian (no protection) limit *κ*/2+*γ*_h_/2+Δ. In the 1% sample, Γ(0)/2*π*=21 GHz is 35 GHz narrower than the Lorentzian limit and also narrower than the FWHM of the initial inhomogeneous broadening of the ensemble, thus indicating that the polariton decay is slower than the decoherence of the initial ensemble. We point out that in Fig. 2c (1% Nd:YVO), Γ/2*π* slightly increases around *δ*=50 GHz before decreasing to a minimum of 21 GHz on resonance. That increase might be explained by coupling to one of the Nd–Nd pair site that is known to be blue-detuned from the central transition by 48 GHz (ref. 24; Supplementary Note 3). The data for the 1% sample is not compared with a theoretical model because, as mentioned above, the exact distribution of ions is unknown. For the 0.1% sample shown in Fig. 2f, Γ(0)/2*π*=22 GHz and the data shows good agreement with the theoretical decay (red curve) for a Gaussian-distributed ensemble with the same FWHM as the joint distribution of the two Zeeman branches (Supplementary Note 2). In the on resonance, strong coupling limit, the theoretical decay is expressed as Γ=*κ*/2+*γ*_h_+*π*Ω^2^*ρ*(Ω) (ref. 15), which reaches the full protection limit of Γ=*κ*/2+*γ*_h_=2*π* × 22 GHz as indicated in Fig. 2f. In our case, the experimental data approached this limit. The residual broadening estimated from the *π*Ω^2^*ρ*(Ω) term was ≈0.1 GHz, more than two orders of magnitude suppressed compared with the case of no protection (where the residual broadening would be 14.6 GHz). Although close to fully protected, the total decay rate was not much slower than the initial ensemble decoherence (FWHM 24 GHz (Δ/2*π*=14.6 GHz) by treating the two Zeeman branches as one joint distribution). To contrast with the case of no protection, we also plot in green the theoretical decays of upper and lower polaritons for a Lorentzian distribution (Supplementary Note 1) assuming the same Δ as for our ensembles. In this case, the atom- and cavity-like polariton widths converge to the Lorentzian limit at zero detuning.

### Time-domain measurement of extended Rabi oscillations

The cavity-protected system acts as a quantum interface where a broadband photon can be transferred to a super-radiant atomic excitation. We measured these coherent, ultra-fast dynamics using pulsed excitations of the polaritons. The experimental set-up is depicted in Fig. 1d. A mode-locked Ti:sapphire laser with a 85 MHz repetition rate (Thorlabs Octavius) was filtered to a pulse width of 4(1.5) ps using a monochromator, which was sufficient to simultaneously excite both upper (denoted by |*ω*_+_>, and referred to as |1> thereafter) and lower (|*ω*_−_>, also referred to as |0> thereafter) polaritions in 0.1% (1%) device. The filtered laser was attenuated and sent through a Michelson interferometer to produce two pulses with less-than-one mean photon number separated by a variable delay 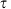. These pulses were coupled into the cavity (red path) and the transmitted signal was collected (blue path) for direct detection using a silicon single-photon counter. The integrated counts at varying delays produce optical field autocorrelation signals revealing the temporal evolution of the polaritons. The mirror at each Michelson arm was interchangeable with a Gires–Tournois Interferometer (GTI) etalon, which generates a ∼*π*/2 phase chirp between the two polaritons (‘Methods' section). Furthermore, a narrow bandpass filter was optionally inserted in either arm that allowed only one polariton to be excited. This combination enabled a comprehensive polariton excitation scheme that covered individual polariton |0> or |1>, and superposition states of two polaritons that is, |+>=1/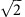
(|0>+|1>) or 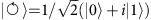.

**Figure 3:**
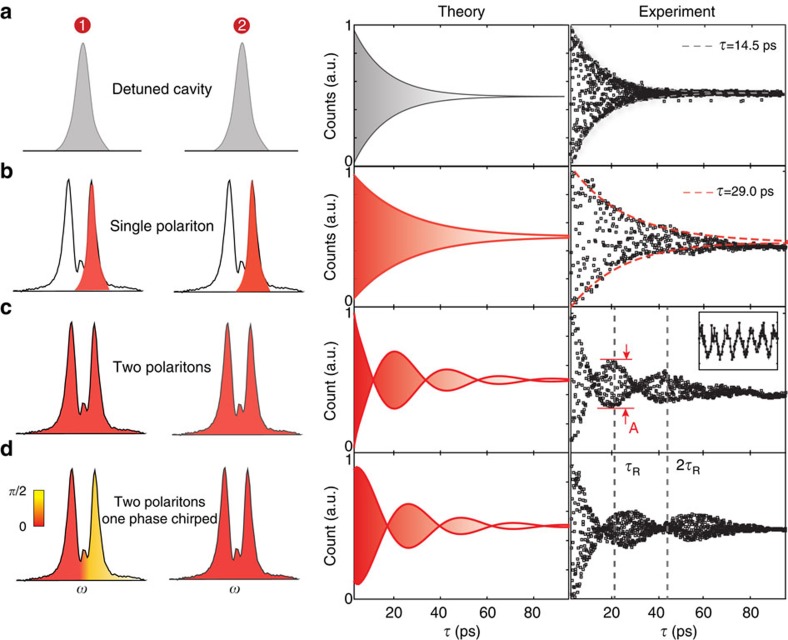
Time-domain interferometric transmission measurements. Left panels show the cavity transmission spectra (black outlines) of the probed system. Coloured areas show the spectral range addressed by the probe pulses (left is first pulse, right is second pulse). Grey is for uncoupled cavity; red for excited polariton states: yellow for the polarition with a shifted phase. (**a**) Simulated and measured cavity decay (that is, lifetime) when uncoupled from the atoms. (**b**) Decay of singly excited lower (upper) polarition (that is, |0>(|1>)). (**c**) Decayed oscillations when both polaritons were excited with transform-limited pulses. (**d**) Decayed and time-shifted oscillations when two polaritons were initially excited with a *π*/2 phase difference (that is, |0>+*i*|1>) by the first pulse and in phase by the second pulse. (**b**–**d**) show extended decays times that are about twice that in **a**, confirming a nearly full protection against ensemble-induced decoherence. The dotted lines mark multiples of Rabi periods 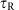. The inset shows a few fine fringes scanned around 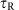.

Figure 3 plots the theoretical interference fringe amplitudes along with the measured results for several two-pulse excitation schemes for the 0.1% cavity in which maximum protection was observed. The results for the 1% device are presented in Supplementary Fig. 3 and Supplementary Note 6. The mean photon number per pulse coupled into the cavity was estimated to be *μ*=0.5. The case of an uncoupled cavity is plotted in Fig. 3a, showing a fitted decay constant (4/*κ*) of ∼14.5 ps (Supplementary Note 5). When only one polariton was excited, the decay was extended to 29.0 ps (Fig. 3b). For the superposition state |+>, Ramsey-like fringes were obtained, revealing extended Rabi oscillations between photonic and atomic excitations beyond the cavity lifetime (Fig. 3c). In the case of Fig. 3d, the first pulse excited the two polaritons with a phase chirp. The resulting fringe showed the Rabi oscillations with the nodes shifted with respect to 3c by about 5.5 ps (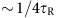), in agreement with the theoretical model (Supplementary Note 4). Those nodes correspond to the quantum excitation being entirely stored in the ensemble with no energy left in the cavity mode, during which time the stored qubit dephases at a significantly slower rate than the inhomogeneous broadening.

### Quantum state transfer with protected Nd ensembles

**Figure 4:**
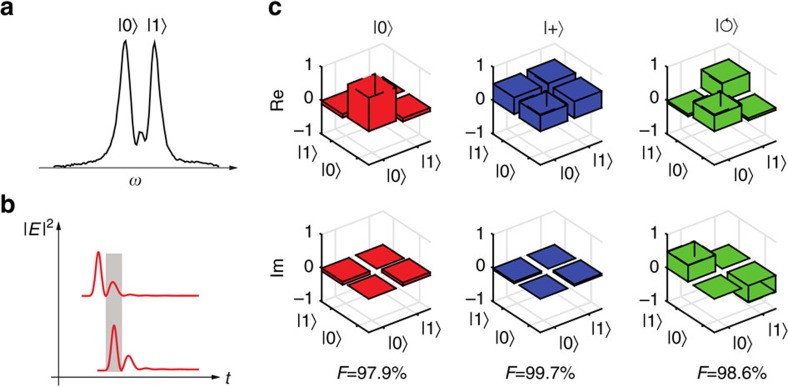
Broadband qubit transfer with protected Nd ensembles. (**a**) Two polaritons serve as eigenbasis for a frequency bin qubit. A phase chirp can be added to construct a generic qubit 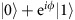. (**b**) Simulated time-domain evolution of the cavity field intensities of two qubit-encoded photons, showing temporal overlap between the retrieved photon 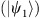 and the second photon (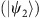) directly transmitted through the cavity at time 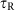. The fields dominantly overlap within a temporal window (grey area) when interference occurs yielding integrated photon counts proportional to the overlap 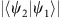. The overlap after the grey window was negligibly small due to fast cavity decays. (**c**) Reconstructed density matrices for each input qubit (top row) delayed by 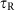 showing high fidelities.

This quantum interface is similar to an AFC with two teeth, one at each polariton, that form the basis of a frequency bin qubit as shown in Fig. 4a. Photons are stored and then released after inverse of the teeth spacing, which is a Rabi period 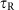. The interface bandwidth is ∼50 GHz, spanning two polaritons, and the qubits are of the form |0>, |+>=1/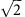
(|0>+|1>) or 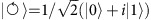. To characterize this process, quantum state tomography on the released qubit after a delay 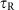 was performed. As direct projection measurements were difficult given the high-bandwidth, we adopted an interferometric scheme (Fig. 4b) to assess the input/output fidelity 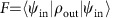, where 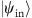 is the input qubit state and *ρ*_out_ is the density matrix for the retrieved state, from a set of fringe signals including those in Fig. 3 (‘Methods' section and Supplementary Note 7). The reconstructed density matrices *ρ*_out_ for |0>, |+>, and 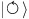 input states along with their respective fidelities are shown in Fig. 4c. A mean fidelity of 98.7±0.3% is obtained, which significantly surpasses the classical fidelity limit (the best qubit input/output fidelity one can achieve using a classical intersect-resend strategy^25^ (Supplementary Note 8)) of 74.9±0.04% that takes into account the Poissonian statistics of the coherent input photons (with *μ*=0.5) and an imperfect but high storage-retrieval efficiency of 25.6±1.2% (‘Methods' section)^26^,^27^. The estimated fidelities take into account imperfections in the preparation and measurement of the qubit, such as leakage of travelling waves through the cavity and inaccurate phase shift (ideally *π*/2) by the GTI etalon. The high fidelity indicates a robust quantum transfer with a bandwidth that is significantly broader than existing rare-earth-based light-matter interfaces, with the highest bandwidth demonstrated so far of 8 GHz in a Erbium doped fibre^28^, and 5 GHz reported in a Thulium doped LiNbO_3_ waveguide^29^. To highlight the benefit of cavity protection, we also evaluated the qubit fidelity at a delay of 2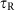, which would be equivalent to the case without cavity protection where the qubit would decohere twice as fast. The measured fidelities at 2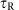 dropped to 83% for |0>, 70% for |+> and 69% for 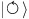, which no longer beats the classical limit.

## Discussion

While this interface efficiently maps the photonic qubit to the ensemble, the qubit dissipates at a rate of *κ*/2. Improvements in the cavity quality factor to state of the art values of *Q*∼10^6^ would achieve storage for 1 ns (enough for perform 50 Rabi flips). The interface with very narrow polariton lindwidths could still map a broadband photon to the ensemble though at the expense of efficiency. To enable long-term storage like in an AFC-spin-wave memory^7^, the qubit can be transferred from the super-radiant state excitation to a long-lived spin level by applying a *π* pulse within 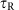 time. Upon recall, another *π* pulse can transfer the qubit back to the polariton states and then a cavity photon. For faithful spin-wave storage, further spectroscopic studies are needed to verify the spin coherence time in the rare-earth ensembles. Also, the Rabi frequency of the driving pulses should exceed the polariton linewidths, which is attainable given the strong light confinement in current nanobeam devices. Compared with existing AFC-spin-wave memories, this interface would not require any preparation steps or time-multiplexing to achieve always-ready operation. Taking advantage of on-chip platforms also allow spatially and temporally multiplexed storage by routing photons to an array of nanocavities with different delays. Most notably, the cavity-protected mapping of a photonic qubit to a collective super-radiant state could compliment the reported coupling of rare-earths to a superconducting resonator^30^ to fulfil efficient quantum transduction between optical and microwave photons via Zeeman or hyperfine transitions in rare-earth ensembles^12^,^13^.

## Methods

### Nanocavity design and characterization

The triangular nanobeam has a width of 770 nm and length of 15 μm. Forty periodic subwavelength grooves of 185 nm along the beam axis were milled on top of the nanobeam. The period of the grooves were modulated at the center of the beam to form defect modes in the photonic bandgap. The fundamental TM mode, with side, top and cross-section views shown in Fig. 1c, is chosen because it aligns with the strongest dipole of the 879.7 nm transition in Nd:YVO. The theoretical quality factor is 300,000 with a mode volume of 1(*λ*/n)^3^ (ref. 22). Transmission of the nanocavity was measured by vertically coupling free-space input into the nanobeam via a 50 × objective lens and a 45°-angled reflector milled into the sample surface, and the cavity output was collected via the other reflector which sent the light back vertically to free space. The output signal was effectively isolated from the input reflections or other spurious light by spatial filtering using a pin hole. When the cavity is on resonance, we measured a total transmission (from free-space input to output) of 20%, which was primarily limited by the imperfect coupling into the nanobeam. The output signal also contained leakage travelling waves (5%) due to finite extinction of the photonic bandgap and other spurious reflections in the system.

### Polariton excitation and frequency qubit preparation

The GTI etalon was made of a 250 *μ*m thick quartz slide with backside coated with a gold film. The front side was uncoated, which had a reflectivity of 4%. This etalon produces a nearly linear dispersion of 4*π*/nm over a free spectral range of 0.5 nm near 880 nm. After the GTI etalon, the transform-limited laser pulse acquired a phase chirp, which excited a mixed polariton state approximated by 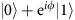, where *φ* is the phase shift over the Rabi splitting. For our custom made etalon, *φ*≈0.52*π* and the corresponding polarition state was close to (|0>+*i*|1>).

### Quantum state tomography based on two-pulse interferometry

The electric field operators for the two consecutive photonic states 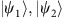 coupled to the cavity are written as 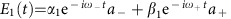 and 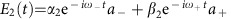, respectively. The field operator at the single-photon detector is 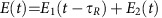 corresponding to the first photon delayed by 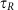 after storage in the ensemble, which interferes with the second photon. The count rate on the detector is 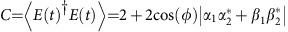, where *φ* is the phase difference between the two photon wavepackets. This gives interference fringes with an amplitude 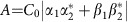 as labelled in Fig. 3c, where *C*_0_ is a constant factor. By encoding a set of four basis states (Pauli tomography basis) on the second photon, that is, *α*_2_|0>+*β*_2_|1>, we construct the set of experimental amplitude parameters *A*_*j*_, *j*=0...3 (*A*_0_ for 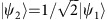; *A*_1_ for 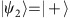, *A*_2_ for 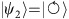; *A*_3_ for 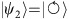) which are analogous to the set of projection measurement outcomes for calculating the density matrix 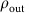 (see Supplementary Note 7 for detailed derivations),


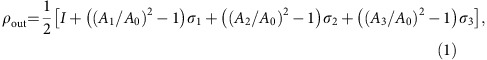


where 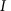 is the identity operator and 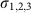 are the Pauli spin operators. Then we perform a maximal likelyhood estimation^31^ to obtain a physical density matrix, which is used to calculate the fidelity 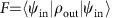


### Qubit storage and retrieval efficiency

The storage efficiency is defined as the probability that a photon in the cavity mode is transferred to a collective excitation in the ensemble. This storage efficiency is intrinsically 100% as the polariton modes under cavity protection condition are maximally entangled states between the cavity and the super-radiant state. The retrieval efficiency is defined by the number of photons emitted at the cavity output during the second Rabi period (grey window in Fig. 4b) versus the total transmitted photons. On the basis of the temporal distribution of the transmitted photons (deconvolved from the oscillation signal in Fig. 3c), the integrated counts during the second Rabi period (grey window in Fig. 4b) was 25.6±1.2% of the total transmitted photons. Note that this storage and retrieval efficiency does not take into account the input coupling and scattering loss. Including the 20±2% transmission efficiency through the device, the overall system efficiency was 5.1±0.7%. The corresponding classical bound for qubit storage fidelity would be 78.9±0.05%, still significantly below the measured fidelities in Fig. 4c.

### Data availability

The data that support the findings of this study are available from the corresponding author on request.

## Additional information

**How to cite this article:** Zhong, T. *et al*. Interfacing broadband photonic qubits to on-chip cavity-protected rare-earth ensembles. *Nat. Commun.* **8,** 14107 doi: 10.1038/ncomms14107 (2017).

**Publisher's note**: Springer Nature remains neutral with regard to jurisdictional claims in published maps and institutional affiliations.

## Supplementary Material

Supplementary Information Supplementary figures, supplementary notes and supplementary references.

Supplementary Movie 1 Cavity transmission spectrum of the 1% Nd:YVO device as the cavity is tuned to atomic ensemble resonance.

Supplementary Movie 2 Cavity transmission spectrum of the 0.1% Nd:YVO device as the cavity is tuned to atomic ensemble resonance.
