# Supplementary material for: Interfacing broadband photonic qubits to on-chip cavity-protected rare-earth ensembles
Source: Nat Commun. 2017 Jan 16;8:14107. doi: 10.1038/ncomms14107 (PMC5241816; doi:10.1038/ncomms14107)
Supplement: Supplementary Information — Supplementary figures, supplementary notes and supplementary references. [file ncomms14107-s1.pdf]

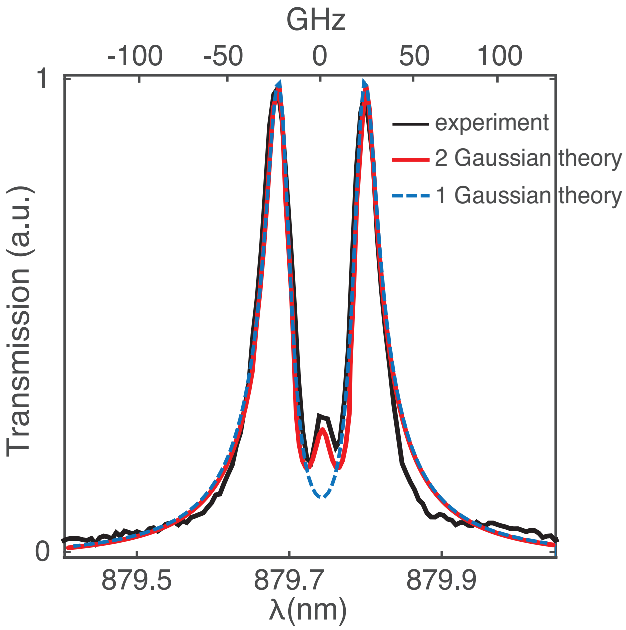


**Supplementary Figure** 1. **Experimental and theoretical on-resonance spectrum in a 0.1% Nd:YVO cavity** Black curve is the experimental spectrum. Red curve is the theoretical spectrum using a model based on two distinct Gaussian sub-ensembles, which reveals a middle peak that is consistent with the experiment. Blue dotted curve is the theoretical spectrum using a model based on one Gaussian distribution whose inhomogeneous width approximates the total width of the two Gaussian sub-ensembles. The blue curve does not show the middle peak, but otherwise coincides with the red curve well.

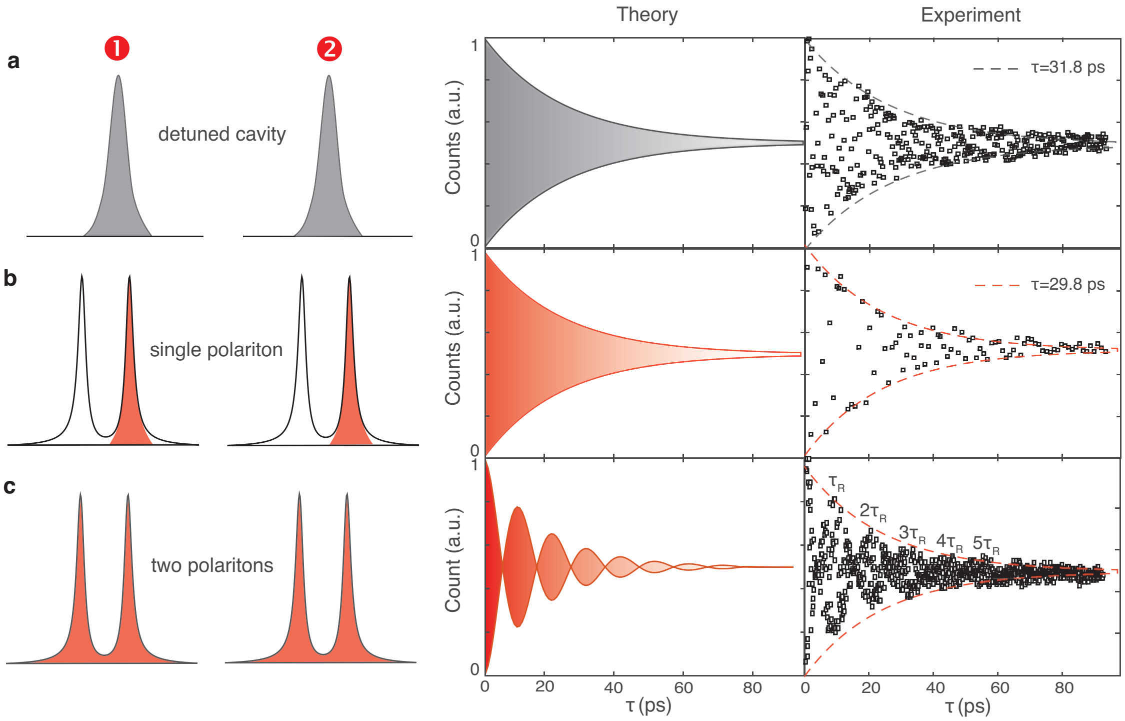


**Supplementary Figure** 2. **Time-domain interferometric measurements of the 1% Nd:YVO cavity transmission.** Left panels show the cavity transmission spectra for the two pulses under different excitation schemes. Grey area is for uncoupled cavity; red for excited polariton states. The colored areas were plotted against the polariton spectrum (solid curve). **a,** Simulated and measured cavity decay (i.e. lifetime) when uncoupled from the atoms. **b,** Decay of singly excited lower (upper) polarition. **c,** Decayed oscillations when both polaritons were excited with transform-limited pulses. The dotted grey and red curves are single exponential fit to the decaying amplitude of the fringe signal, from which the decay time constants are extracted.

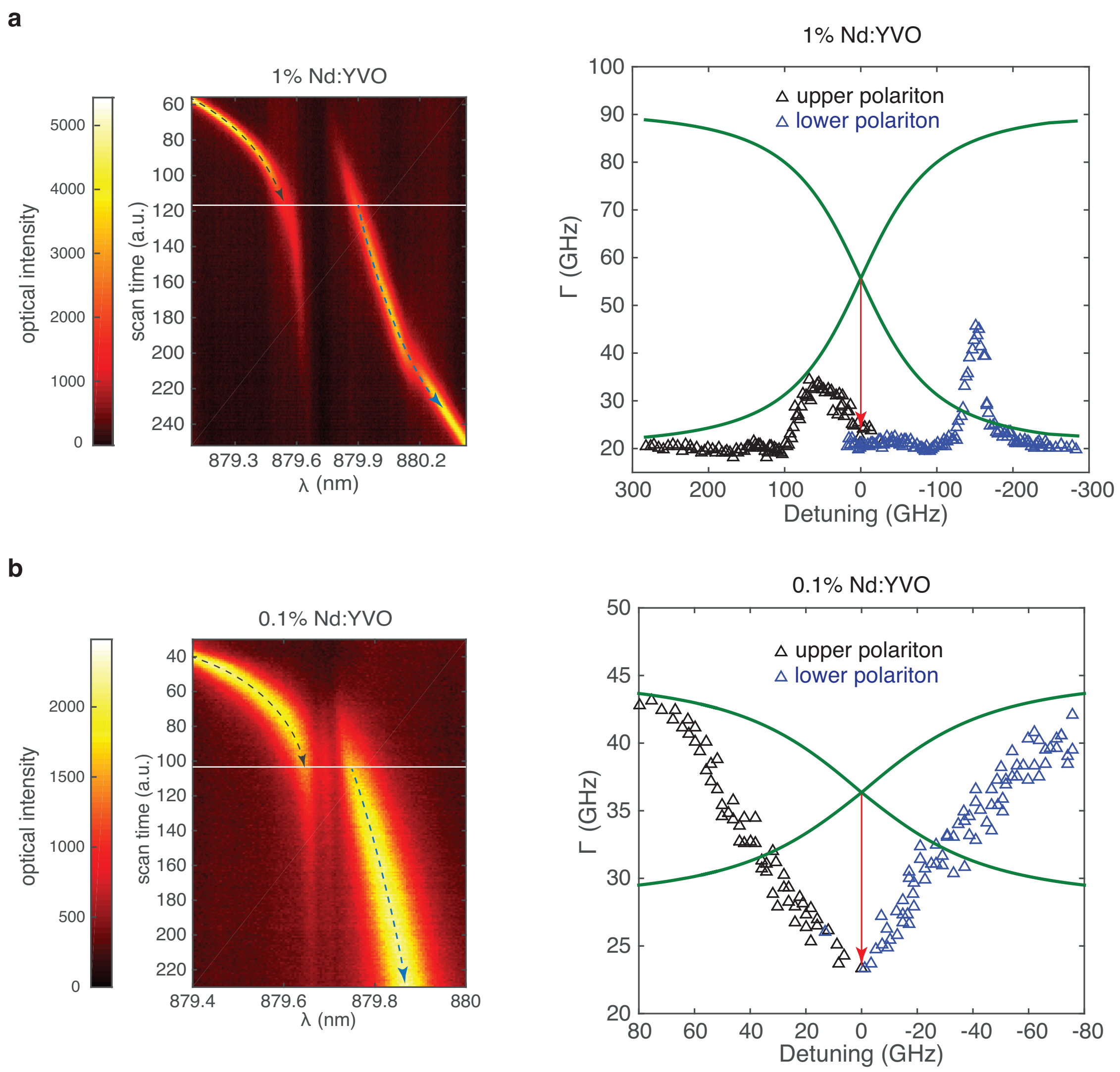


**Supplementary Figure** 3. **Experimental polariton linewidths versus detuning. a,** 1% Nd:YVO. **b,** 0.1% Nd:YVO. Black (dark blue) triangles are for lower (upper) polariton. Green curves are theoretical linewidths for both polaritons assuming a Lorentzian ensemble distribution.

## Supplementary Note 1. Theoretical decay rates of a coupled cavity-ensemble system

The cavity-ensemble coupled system is described by the Tavis-Cummings Hamiltonian. We model the system following [1], which consists of a cavity mode $a$ of frequency $\omega_0$ coupled with strength $g_k$ to a distribution of $N$ two-level emitters described by modes $b_k$. We define the cavity frequency $\omega_0$ and the frequency of each emitter as $\omega_k$. We account for the atomic dephasing rate (homogeneous linewidth) $\gamma_h$ and label the cavity intensity decay as $\kappa$. Using the standard input-output formalism for a two sided cavity with input field $c_{in}$, reflected field $c_r$, and transmitted field $c_t$ gives the Heisenberg equations for the system:

$$\begin{aligned}
\dot{a} &= -\left[\frac{\kappa}{2} + i(\omega_0 - \omega)\right] a - \sqrt{\frac{\kappa}{2}} c_{in} + \sum_k g_k b_k \\
\dot{b}_k &= -\left[\frac{\gamma_h}{2} + i(\omega_k - \omega)\right] b_k - g_k a \\
c_t &= \sqrt{\frac{\kappa}{2}} a \\
c_r &= c_{in} + \sqrt{\frac{\kappa}{2}} a.
\end{aligned} \tag{1}$$

By solving this set of equations in the steady state, we arrive at the complex transmission of the cavity:

$$t(\omega) = \frac{\langle c_t \rangle}{\langle c_{in} \rangle} = \frac{-\frac{\kappa}{2i}}{\omega_0 - \frac{i\kappa}{2} - \omega - \sum_k \frac{g_k^2}{\omega_k - \frac{i\gamma_h}{2} - \omega}}. \tag{2}$$

For a large number of emitters, we can define the distribution of emitters in terms of a continuous spectral density $\rho(\omega) = \sum_k \frac{g_k^2 \delta(\omega - \omega_k)}{\Omega^2}$. Here $\Omega$ is the collective coupling strength defined by $\Omega^2 = \sum_k g_k^2$. The continuum form of the transmission is then [1]

$$t(\omega) = \frac{-\frac{\kappa}{2i}}{\omega_0 - \frac{i\kappa}{2} - \omega + \Omega^2 \int \frac{\rho(\omega') d\omega'}{\omega - \omega' + \frac{i\gamma_h}{2}}}. \tag{3}$$

**Lorentzian distribution** In the case of a Lorentzian distribution, $\rho(\omega) = \frac{\Delta}{\pi} \frac{1}{\Delta^2 + (\omega - \omega_a)^2}$, where $2\Delta$ is the FWHM of the inhomogeneous linewidth and $\omega_a$ denotes the center frequency of the ensemble, Eq. (3) is integrable to

$$t(\omega)_{\text{Lorentzian}} = \frac{-\frac{\kappa}{2i}}{\omega_0 - \frac{i\kappa}{2} - \omega + \frac{\Omega^2}{\omega - \omega_a + i\gamma_h/2 + i\Delta}}. \tag{4}$$

The poles of the transmission function yield

$$\omega_\pm = \omega_a + \delta/2 - i\frac{\kappa + \gamma_h + 2\Delta}{4} \pm \sqrt{\Omega^2 + [(i\kappa - 2i\Delta - i\gamma_h - 2\delta)/4]^2}, \tag{5}$$

where we define the cavity-ensemble detuning as $\delta = \omega_0 - \omega_a$. Eq. (5) determines the locations and linewidths of the two polariton modes:

$$\Gamma_\pm = \frac{\kappa + \gamma_h + 2\Delta}{2} \pm \mathrm{Im}(\sqrt{4\Omega^2 + [(i\kappa - 2i\Delta - i\gamma_h - 2\delta)/2]^2}), \tag{6}$$

which checks $\Gamma = \frac{\kappa+\gamma_h+2\Delta}{2}$ for the on-resonance ($\delta$ =0), strong coupling ($\Omega \gg \kappa, \Delta$) condition. The green curves in Fig. 2**c**,2**f** are thereby evaluated from Eq. (6).

**Gaussian distribution** In the case of a Gaussian distribution, $\rho(\omega) = \frac{1}{\Delta\sqrt{\pi}} e^{-(\omega-\omega_a)^2/\Delta^2}$. Note that the definition of $\Delta$ here is different from that in [1]. We define $\Delta$ such that the FWHM of the Gaussian distribution is $2\sqrt{\ln 2}\Delta$, which is consistent with the q-Gaussian definition in the text. Integrating Eq. (2) gives

$$t(\omega,\delta)_{\mathrm{Gaussian}} = \frac{-\frac{\kappa}{2i}}{\omega_a + \delta - \frac{i\kappa}{2} - \omega - i\frac{\Omega^2}{\Delta}\sqrt{\pi} e^{-(\frac{\omega-\omega_a+i\gamma_h/2}{\Delta})^2} \mathrm{erfc}(-i\frac{\omega-\omega_a+i\gamma_h/2}{\Delta})}. \tag{7}$$

where erfc is the complex complementary error function. There is no straightforward analytical solution for the poles of the transmission function. Therefore we numerically solve for the transmission poles and the polariton linewidth as a function of the cavity-ensemble detuning $\delta$.

To plot the theoretical decay rates in Fig. 2**c**,2**f**, we need experimental values for $\kappa$, $\Delta$, $\Omega$ and $\gamma_h$. In the case of a far detuned cavity, using a spectrometer (with resolution of 4 GHz) we directly measured $\kappa = 2\pi\times44$ GHz for the 0.1% Nd:YVO device, and $\kappa = 2\pi\times20$ GHz for the 1% Nd:YVO device. As discussed in the main text, the atomic distributions are approximately Gaussian, with $\Delta/2\pi$ =14.6 GHz from fitting the two branches with one Gaussian for the 0.1% Nd:YVO device, and a $\Delta/2\pi$=45.6 GHz for the 1% Nd:YVO device. We then measured $\gamma_h/2\pi$=1/$\pi T_2$= 0.82 MHz for 0.1% Nd:YVO using two-pulse photon echoes. We could not measure any echo signal from the 1% Nd:YVO device, which is probably due to significant ion-ion interactions in a highly doped sample. However, we estimated an upper bound of $\gamma_h/2\pi \leq$ 40MHz by transient hole burning in the 1% sample. To determine $\Omega$, we fit the on-resonance transmission spectra (Fig. 2**b,e**) with $\Omega$ as the only free parameter. Fitting based on Eq. (7) allows us to determine the collective coupling strength $\Omega = 2\pi\times25$ GHz for the 0.1% device, and $\Omega = 2\pi\times55$ GHz for the 1% device.

### Supplementary Note 2. Explanation of the middle peak in 0.1% Nd:YVO nanocavity

When tuning the cavity across the Nd $Y_1$-$Z_1$ transition in the 0.1% device, we observed a weak transmission peak between the two polariton modes. This peak is caused by cou-

pling a cavity to two distinct ensembles of emitters spectrally separated by $2\delta_a$, which was theoretically predicted and analysed in Section VI. B of [1]. This middle peak corresponds to the eigenstate resulting from the coupling between the cavity mode and the antisymmetric state $|A\rangle = (|G_1, S_2\rangle - |S_1, G_2\rangle)/\sqrt{2}$, where $G_j, S_j$ are ground and excited state of the superadiance collective state of $j$th ensemble. According to [1], the state giving rise to the middle peak is written as $i\delta_a|1, G_1, G2\rangle + \Omega\sqrt{2}|0, A\rangle$. This state would be completely dark if $\Omega \gg \delta_a$, and has a small cavity component otherwise, which we show below is the case in our 0.1% Nd:YVO device.

To model the system, we start with a atomic distribution as a summation of two Gaussian distributions spectrally separated by $2\delta_a$: $\rho(\omega) = \frac{1}{2\Delta\sqrt{\pi}}e^{-(\omega-\omega_a-\delta_a)^2/\Delta^2} + \frac{1}{2\Delta\sqrt{\pi}}e^{-(\omega-\omega_a+\delta_a)^2/\Delta^2}$. Each Gaussian subensemble is one Zeeman branch and consists of half of the total population due to a thermal distribution at 3.6 K. The on-resonance ($\delta = 0$) cavity transmission spectrum can be obtained by evaluating Eq. (3), which modifies Eq. (7) to

$$t(\omega) = \frac{-\frac{\kappa}{2i}}{\omega_a - \frac{i\kappa}{2} - \omega - i\frac{\Omega^2}{2\Delta}\sqrt{\pi}\sum_{j=1,2} e^{-(\frac{\omega-\omega_{aj}+i\gamma_h/2}{\Delta})^2}\mathrm{erfc}(-i\frac{\omega-\omega_{aj}+i\gamma_h/2}{\Delta})}. \tag{8}$$

where $\omega_{a1} = \omega_a - \delta_a$, $\omega_{a2} = \omega_a + \delta_a$ are the central frequencies of two Zeeman branches, respectively.

From the absorption spectrum in Fig. 1**b** for 0.1% Nd:YVO, we extract the parameters $\delta_a$ =8.5 GHz, and $\Delta$ =5.0 GHz. With $\kappa$ and $\gamma_h$ measured above, we plot in Fig. 2**e** and Supplementary Figure 1 the theoretical transmission (red curve) based on Eq. (8). Note that this theoretical curve is not a fit, which clearly reveals the middle peak. The measured transmission spectrum is overlaid with the theory curve, showing good agreement.

In Fig. 2f of the main text, the polariton decay $\Gamma$ was compared to the expected decay from a Gaussian ensemble that has the same FWHM as the sum of the two Zeeman branches combined. Here we justify that this approximation of two Zeeman sub-ensembles by one broader Gaussian distribution is valid. As mentioned in the main text, to treat the entire ensemble as one Gaussian distribution, we find the effective FWHM to be 24 GHz, and $\Delta/2\pi$=14.6 GHz. We plot the expected on resonance transmission using Eq. (7) in blue in Supplementary Figure 1. We see a high degree of agreement between the red and blue curves except for the middle peak region. This means the one-Gaussian approximation captures all the essential properties of the polariton spectrum. This is expected from the conclusions

drawn from [1], that the polariton linewidths only depend on the profile of the tails of the distribution, but not the central region of the distribution function $\rho(\omega)$. Thus we confirm the validity of the one-Gaussian approximation.

### Supplementary Note 3. Experimental polariton linewidths versus detuning

Here we plot the linewidths of both upper (shorter wavelength) and lower (longer wavelength) polaritons as the cavity was tuned from shorter to longer wavelengths across the atomic transition. For 1% Nd:YVO, the evolution of each polariton linewidth with detuning is asymmetric about the ensemble center frequency (Supplementary Figure 2a). Two peaks at 50 GHz, and -160 GHz detuning are evident. We believe these two peaks are due to cavity coupling to two satellite lines of the main transition. Given the high density of this sample, we expect that these satellite lines correspond to the Nd-Nd pair site (resulting from Dzyaloshisky-Moriya interactions), which were measured to be +48 GHz and -166 GHz detuned from the line center [2]. On the other hand, in 0.1% Nd:YVO (Supplementary Figure 2b), the linewidths of upper and lower polaritons as a function of detuning appear to be symmetric. This is expected from the symmetric shape of the ensemble distribution (Fig. 1b in the main text), and the absence of strong pair-site satellite lines at this lower doping concentration. Again, we plot in dark green the theoretical linewidths of the polaritons for the case of a Lorentzian distribution. At zero detuning, the linewidths of both polaritons should converge to a value $\kappa/2 + \gamma_h/2 + \Delta$ (i.e. the Lorentzian limit with no protection effect). In Supplementary Figure 2a, 2b, the gap between the experimental zero-detuning (on-resonance) linewidths and the Lorentzian limit represents the amount of linewidth narrowing and the extent of cavity protection. This narrowing is highlighted by a red arrow.

### Supplementary Note 4. Simulation of the dynamics of the coupled system

To simulate the temporal dynamics of the system (Fig. 3 theoretical plots and Fig. 4b), we start from the discrete form of the differential equations Eq. (1). In this case, simulating the entire system of $N$ emitters entails solving a set of $N \sim 10^6$ coupled differential equations. To make the problem more computationally tractable, we instead solve the system of $N_{sim} \ll N$ coupled emitters with their frequencies randomly assigned according to the experimentally

measured atomic distribution $\rho(\omega)$. The coupling strength of each emitter is set as $g = \Omega/\sqrt{N_{sim}}$ such that the collective coupling strength is held constant at the experimental value $\Omega$ =25 GHz. This reduced set of equations was then solved numerically in Mathematica using the built-in differential equation solver (NDSolve). The number of simulated emitters was increased until the solution converged (i.e. until increasing the number of simulated emitters no longer had an effect on the solution). All emitters were assumed to start in the ground state in an empty cavity. The cavity and probe were assumed to be on resonance with the center of the emitter spectral distribution. The input field consisted of two 4(1.5) ps pulses for 0.1% (1%) doped cavity separated by variable time $\tau$. The amplitude of the integrated interference (corresponding to the measurement) for each value of $\tau$ was determined by integrating the cavity transmission over the simulation time (10 cavity lifetimes).

## Supplementary Note 5. Relationship between the decay time constants of the interferometric signal and the polariton linewidth

The experiments we performed to obtain Fig. 3 are optical field autocorrelation measurements at the single photon level. Here we clarify how the decay times of the interferometric signals (Fig. 3 right panel) are related to the actual polariton decay times. Given a polariton linewidth $\Gamma$, the $1/e$ intensity decay constant is $1/\Gamma$ [3]. The field decay constant would be twice as long $2/\Gamma$. The interference of two identical but time-delayed field generates an autocorrelation function of the field. In our case, the field is exponentially decaying in time. The autocorrelation of an exponential decay function is another exponential decay with twice the decay constant. Therefore, the interference signal should yield a decay constant $4/\Gamma$. We verify this relationship experimentally by measuring the decay time of an empty (far-detuned) cavity. For 0.1% Nd:YVO cavity, $\kappa = 2\pi\times 44$ GHz, thus the cavity lifetime is $1/\kappa$ =3.6 ps. By using the optical autocorrelation technique, we measured a decay constant of the interference signal to be 14.5 ps, as shown in blue-dotted fit in Fig. 3**a**, which is equal to $4/\kappa$.

**Supplementary Note 6. Temporal measurements of the cavity transmission for 1% Nd:YVO device**

Supplementary Figure 3 plots the theoretical interference fringe amplitudes along with the measured results. The mean photon number per pulse coupled in the cavity was estimated to be $\mu$=0.5. The case of an uncoupled cavity is plotted in Supplementary Figure 3a, showing a fitted decay constant ($4/\kappa$) of $\sim$31.8 ps. When only one polariton ($|0\rangle$ or $|1\rangle$) was excited, the decay was 29.8 ps (Supplementary Figure 3b). For the superposition of two polaritons, Ramsey-like fringes were obtained, revealing at least 5 Rabi oscillations (Supplementary Figure 3c). The dotted blue and red curves are single exponential fits to the decaying amplitude of the fringe signal.

**Supplementary Note 7. Construction of qubit density matrix from interference fringes of two photons**

The state of an arbitrary qubit $|\psi\rangle = \alpha|0\rangle + \beta|1\rangle$, where $|\alpha|^2 + |\beta|^2 = 1$, can be determined by taking a set of four projection measurements represented by the operators $\hat{\mu_0} = |0\rangle\langle 0| + |1\rangle\langle 1|$, $\hat{\mu_1} = |+\rangle\langle +|$, $\hat{\mu_2} = |\circlearrowleft\rangle\langle\circlearrowleft|$, $\hat{\mu_3} = |0\rangle\langle 0|$ [4]. The outcome of these measurements are

$$n_j = C\mathrm{Tr}\{\hat{\rho}\hat{\mu_j}\}, \tag{9}$$

where $\rho = |\phi\rangle\langle\phi|$ for a pure state and the scaling factor $C$ is the number of detected photons, which will be set to 1 in the following derivation. We explicitly write out the four $n_j$ values for an arbitrary qubit as

$$\begin{aligned} n_0 &= 0.5(|\alpha|^2 + |\beta|^2) = 0.5 \\ n_1 &= 0.5|\alpha + \beta|^2 \\ n_2 &= 0.5|\alpha - i\beta|^2 \\ n_3 &= |\alpha|^2. \end{aligned} \tag{10}$$

From these values, the four Stokes parameters are calculated as

$$
\begin{aligned}
S_0 &= 2n_0 \\
S_1 &= 2(n_1 - n_0) \\
S_2 &= 2(n_2 - n_0) \\
S_3 &= 2(n_3 - n_0).
\end{aligned} \tag{11}
$$

The density matrix $\hat{\rho}$ is then constructed from the Stokes parameters by

$$\hat{\rho} = \frac{1}{2}\sum_{j=0}^{3}\frac{S_j}{S_0}\hat{\sigma}_j, \tag{12}$$

where $\hat{\sigma}_0$ is the identity operator $\hat{I}$ and $\hat{\sigma}_{1,2,3}$ are the Pauli spin operators.

Now we turn to the measurement of interference between two photonic states represented by the electric field operators $\hat{E}_1(t) = \alpha_1 e^{-i\omega_- t}a_- + \beta_1 e^{-i\omega_+ t}a_+$ and $\hat{E}_2(t) = \alpha_2 e^{-i\omega_- t}a_- + \beta_2 e^{-i\omega_+ t}a_+$, where $\omega_-$, $\omega_+$ are the optical frequencies of the lower and upper polaritons, respectively, with frequency difference $\omega_+ - \omega_- = \Omega_R$. For simplicity, we do not include the finite linewidth of each polariton as it does not affect the results of the tomography measurement. The field operator at the single photon detector is $\hat{E}(t) = \hat{E}_1(t - \tau_R) + \hat{E}_2(t)$ corresponding to the first photon delayed by $\tau_R$ after storage in the ensemble. The count rate on the detector is

$$
\begin{aligned}
C &\propto \langle E(t)^\dagger E(t)\rangle \\
&= 2 + 2\cos\phi|\alpha_1\alpha_2^* + \beta_1\beta_2^* e^{-i\Omega_R\tau_R}| \\
&= 2 + 2\cos\phi|\alpha_1\alpha_2^* + \beta_1\beta_2^*|,
\end{aligned} \tag{13}
$$

where the last equality holds for $\Omega_R\tau_R = 2\pi$. Here $\phi$ is the carrier phase difference between the two photons, which is varied from 0 to $2\pi$ to produce interference fringes with a peak-to-peak amplitude $C_{\max} - C_{\min} = 4|\alpha_1\alpha_2^* + \beta_1\beta_2^*|$. We define a set of experimentally measurable fringe amplitude parameters $A = C_0|\alpha_1\alpha_2^* + \beta_1\beta_2^*|$, where $C_0$ is a constant factor representing the integrated counts. These parameters closely resemble the projection measurement outcomes in Eq. (10) (different by a power of 2) depending on the states encoded on the second photon, i.e. $\alpha_2|0\rangle + \beta_2|1\rangle$. For instance, if we encode the second photon in the same qubit as the first photon but attenuate the intensity by a factor of 2 , i.e. $\alpha_2 = \alpha_1/\sqrt{2}$, $\beta_2 = \beta_1/\sqrt{2}$, we get the amplitude $A_0 = \frac{C_0}{\sqrt{2}}(|\alpha_1|^2 + |\beta_1|^2)$. For $\alpha_2 = \beta_2 = 1/\sqrt{2}$, $A_1 = C_0/\sqrt{2}|\alpha_1 + \beta_1|$. For $\alpha_2 = -i\beta_2 = 1/\sqrt{2}$, $A_2 = C_0/\sqrt{2}|\alpha_1 - i\beta_1|$. For $\alpha_2 = 1$, $\beta_2 = 0$, $A_3 = C_0|\alpha_1|$. Based on

the four amplitude values, we construct an equivalent set of Stokes parameter $S_j^A$

$$\begin{aligned} S_0^A &= 2A_0^2 \\ S_1^A &= 2A_1^2 - 2A_0^2 \\ S_2^A &= 2A_2^2 - 2A_0^2 \\ S_3^A &= 2A_3^2 - 2A_0^2, \end{aligned} \tag{14}$$

from which the density matrix is calculated by

$$\begin{aligned} \hat{\rho} &= \frac{1}{2}\sum_{j=0}^{3}\frac{S_j^A}{S_0^A}\hat{\sigma}_j \\ &= \frac{1}{2}[\hat{I} + \left((A_1/A_0)^2 - 1\right)\hat{\sigma}_1 + \left((A_2/A_0)^2 - 1\right)\hat{\sigma}_2 + \left((A_3/A_0)^2 - 1\right)\hat{\sigma}_3]. \end{aligned} \tag{15}$$

Then we perform a maximal likelyhood estimation [4] to obtain a physical density matrix, which is used to calculate the fidelity $F = \langle\psi_{\rm in}|\hat{\rho}|\psi_{\rm in}\rangle$

**Supplementary Note 8. Classical storage fidelity for weak coherent photons**

The classical fidelity for any storage device measures the best input/output fidelity one can achieve using a classical method. For a given photon number of the input state $N_{\rm ph}$, the maximum classical fidelity is known to be $F = \frac{N_{\rm ph}+1}{N_{\rm ph}+2}$ [5]. For an input pulse that is in a coherent state with a mean photon number $\mu$, the Poissonian statistics give a $N$-photon probablity of $P(N_{\rm ph}) = e^{-\mu}\frac{\mu^{N_{\rm ph}}}{N_{\rm ph}!}$. Accounting for each $N$-photon component, the classical fidelity of a coherent state is then

$$F = \sum_{N_{\rm ph}\geq 1}^{\infty}\frac{N_{\rm ph}+1}{N_{\rm ph}+2}\frac{P(N_{\rm ph})}{1-P(0)} \tag{16}$$

In addition, for an imperfect memory with storage and retrieval efficiency $\eta < 1$, the classical fidelity would be higher because a classical memory can preferentially measure the higher photon component of the input and send out a new qubit. We follow the strategy in [6, 7] that there exists a threshold photon number $N_{\rm min}$ that the classical memory sends out a qubit when the input photon number is greater than this value, which happens with a probability $1-p$. Otherwise the memory sends out a result for input photon $N_{\rm min}$ with probability $p$. Combing the two cases, the memory efficiency is expressed as

$$\eta = \frac{p + \sum_{N_{\rm ph}\geq N_{\rm min+1}} P(N_{\rm ph})}{1-P(0)} \tag{17}$$

For a given $\mu$ and $\eta$, the value of $N_{\text{min}}$ can be readily calculated according to [7],

$$N_{\text{min}} = \min i : \sum_{N_{\text{ph}} \geq i+1} P(N_{\text{ph}}) \leq (1 - P(0))\eta, \tag{18}$$

which is used to obtain the final classical fidelity

$$F_{\text{class}} = \frac{\frac{N_{\text{min}}+1}{N_{\text{min}}+2} p + \sum_{N_{\text{ph}} \geq N_{\text{min}+1}} \frac{N_{\text{ph}}+1}{N_{ph}+2} P(N_{\text{ph}})}{\eta(1 - P(0))} \tag{19}$$

**Supplementary References**


[1] Diniz, I. *et al.* Strongly coupling a cavity to inhomogeneous ensembles of emitters: Potential for long-lived solid-state quantum memories. *Phys. Rev. A* **84,** 063810 (2011).

[2] Laplane, C., Cruzeiro, E. Z., Fröwis, F., Goldner, P., & Afzelius, M. High precision measurement of the Dzyaloshinsky-Moriya interaction between two rare-earth ions in a solid. *Phys. Rev. Lett.* **117,** 037203 (2016). (2016).

[3] Takahashi, Y., Hagino, H., Tanaka, Y., Song, B.-S., Asano, T., & Noda, S. High-Q nanocavity with a 2-ns photon lifetime. *Opt. Express* **15,** 17206-17213 (2007).

[4] James, D. F. V., Kwiat, P. G., Munro, W. J., & White A. G. Measurement of qubits. *Phys. Rev. A* **64,** 052312 (2001).

[5] Massar, S., & Popescu, S. Optimal Extraction of Information from Finite Quantum Ensembles. *Phys. Rev. Lett.* **74,** 1259 (1995).

[6] Specht, H. P., Nölleke, C., Reiserer, A., Uphoff, M., Figueroa, E., Ritter, S., & Rempe, G. A single-atom quantum memory. *Nature* **473,** 190-193 (2011).

[7] Gündogan, M., Ledingham, P. M., Almasi, A., Cristiani, M., & de Riedmatten, H. Quantum storage of a photonic polarization qubit in a solid. *Phys. Rev. Lett.* **108,** 190504 (2012).
